# Characteristics and outcomes of peer consultations for assisted dying request assessments: Cross-sectional survey study among attending physicians

**DOI:** 10.3389/fpubh.2023.1100353

**Published:** 2023-03-29

**Authors:** Stijn Vissers, Sigrid Dierickx, Luc Deliens, Freddy Mortier, Joachim Cohen, Kenneth Chambaere

**Affiliations:** ^1^End-of-Life Care Research Group, Vrije Universiteit Brussel (VUB) and Universiteit Gent, Brussels, Belgium; ^2^Department of Public Health and Primary Care, Universiteit Gent, Ghent, Belgium; ^3^Bioethics Institute Ghent, Department of Philosophy and Moral Sciences, Universiteit Gent, Ghent, Belgium

**Keywords:** assisted dying, euthanasia, physicians, medical end-of-life practice, peer consultation, medical decision-making, end-of-life care, Belgium

## Abstract

**Background:**

In most jurisdictions where assisted dying practices are legal, attending physicians must consult another practitioner to assess the patient's eligibility. Consequently, in some jurisdictions, they can rely on the expertise of trained assisted dying consultants (trained consultants). However, these peer consultations remain under-researched. We examined the characteristics and outcomes of peer consultations to assess an assisted dying request with trained consultants, and explored how these characteristics influence the performance of assisted dying.

**Methods:**

We conducted a cross-sectional survey in 2019–2020 in Belgium among attending physicians who had consulted a trained consultant for an assisted dying request assessment (*N* = 904).

**Results:**

The valid response rate was 56% (502/903). The vast majority of attending physicians (92%) who had consulted a trained consultant were general practitioners. In more than half of the consultations (57%), the patient was diagnosed with cancer. In 66%, the patient was aged 70 or older. Reported as the patients' most important reasons to request assisted dying: suffering without prospect of improving in 49% of the consultations, loss of dignity in 11%, pain in 9%, and tiredness of life in 9%. In the vast majority of consultations (85%), the attending physician consulted the trained consultant because of the expertise, and in nearly half of the consultations (46%) because of the independence. In more than nine out of ten consultations (91%), the consultant gave a positive advice: i.e., substantive requirements for assisted dying were met. Eight out of ten consultations were followed by assisted dying. The likelihood of assisted dying was higher in consultations in which loss of dignity, loss of independence in daily living, or general weakness or tiredness were reasons for the request.

**Conclusion:**

Our findings indicate that the peer consultation practice with trained consultants is most often embedded in a primary care setting. Moreover, our study corroborates previous research in that assisted dying is performed relatively less frequently in patients with cancer and more often in patients with general deterioration. Our findings suggest that attending physicians hold peer consultations with trained consultants to endorse their own decision-making and to request additional support.

## 1. Introduction

In the last decade, assisted dying—i.e., intentionally assisting in ending the life of a competent person (further referred to as the “patient”) at his or her own explicit and voluntary request by means of lethal drugs—has become an increasingly prevalent practice in and across several jurisdictions (1). Assisted dying comprises the practices of euthanasia and physician-assisted suicide (P.A.S.). In euthanasia, health practitioners themselves administer the lethal drugs. In P.A.S., health practitioners provide or prescribe the lethal drugs to patients who then self-administer them. With Spain and New Mexico (U.S.A.) being the latest jurisdictions to have enacted assisted dying legislation (2, 3), nearly 300 million people across the globe—i.e., about 4% of the world population- are currently living in jurisdictions where assisted dying is lawful (1). That number will likely increase, since other jurisdictions are debating the enactment of assisted dying legislation—for example, Scotland and several U.S. states including Florida, New York, and Arizona (3, 4). Consequently, assisted dying has, or will, become an important part of medical practice and health care systems in these societies.

Almost all jurisdictions with assisted dying legislation have put in place legal requirements that must be properly assessed and met before assisted dying can be carried out (1). These requirements relate to eligibility criteria for the person requesting assisted dying (such as health condition) and procedural due care criteria such as peer consultation and reporting procedures. The present study was undertaken in Belgium, where euthanasia is legally regulated while the legal status of physician-assisted suicide remains unclear. The Belgian Federal Control and Evaluation Commission for Euthanasia (FCECE) and the Belgian National Board of Physicians treat P.A.S. as a form of euthanasia under certain conditions (5, 6). The FCECE reviews physician-assisted suicide cases on the basis of the legal requirements for euthanasia cases (6), which are listed in Box 1.

Box 1 **Legal requirements stipulated in the Belgian Act on Euthanasia (7).**

Euthanasia is defined as intentionally terminating life by someone other than the person concerned, at the latter's request.

- The physician who performs euthanasia commits no criminal offense when he or she ensures that

◦ The patient has attained the age of majority and is legally competent and conscious at the moment of making the request^*^;
◦ The request is voluntary, well-considered and repeated, and is not the result of any external pressure;
◦ THE patient is in a medically futile condition of constant and unbearable physical or mental suffering that cannot be alleviated, resulting from a serious and incurable disorder caused by illness or accident;
◦ He or she adheres to the conditions and procedures as provided in this Act on Euthanasia.

- Without prejudice to any additional conditions imposed by the physician on his/her own action, before carrying out euthanasia he/she must in each case:

◦ Inform the patient about his or her health condition and life expectancy, discuss with the patient his/her request for euthanasia and the possible therapeutic and palliative courses of action and their consequences. Together with the patient, the physician must come to the belief that there is no reasonable alternative to the patient's situation and that the patient's request is completely voluntary;
◦ Be certain of the patient's constant physical or mental suffering and of the durable nature of his/her request. To this end, the physician has several conversations with the patient spread out over a reasonable period of time, taking into account the progress of the patient's condition;
◦ Consult another physician about the serious and incurable character of the disorder and inform him/her about the reasons for this consultation. The physician consulted reviews the medical record, examines the patient and must be certain of the patient's constant and unbearable physical or mental suffering that cannot be alleviated. The physician consulted reports on his or her findings. The physician consulted must be independent of the patient as well as of the attending physician and must be competent to give an opinion about the disorder in question. The attending physician informs the patient about the results of this consultation;
◦ If there is a nursing team that has regular contact with the patient; discuss the request of the patient with the nursing team or its members;
◦ If the patient so desires, discuss his or her request with relatives appointed by the patient;
◦ Be certain that the patient has had the opportunity to discuss his/her request with the persons that he or she wanted to meet.

- The patient's request must be in writing.
- After performing euthanasia, the attending physician must notify the case for review to the Belgian Federal Control and Evaluation Commission for Euthanasia (FCECE). Subsequently, the FCECE assesses whether the euthanasia case complies with the substantive and procedural requirements stipulated in the Belgian Act on Euthanasia.

^*^As from 2014, competent minors with capacity of discernment can also receive euthanasia in the case of unbearable physical suffering that cannot be alleviated, resulting from a serious, incurable condition caused by illness or accident that will lead to death within a short period of time. Moreover, the attending physician needs to seek advice on the legal eligibility from a pediatric psychiatrist or psychologist, and the consent of the patient's legal representatives.

Peer consultation with another independent health practitioner is a procedural requirement incorporated into nearly all assisted dying legislation (1). This independent health practitioner, or consultant, must be a physician (1). In some provinces in Canada, however, the consultant may also be a nurse practitioner. Although legal modalities of peer consultation differ across jurisdictions, the common principle implies that the attending health practitioner must consult with an independent peer practitioner, or consultant, who must assess the patient's eligibility for assisted dying. This results in either a positive or negative advice from the consultant: i.e., the patient is either eligible or not for assisted dying. Consequently, peer consultation represents a due care or due diligence practice to safeguard patients, since consultants may identify those persons who are not eligible for assisted dying. Therefore, peer consultation practice is also considered an essential control measure within assisted dying practice. However, in some jurisdictions—Belgium and the Netherlands, for example—attending physicians are not legally obliged to adhere to the advice of the consultant and can perform assisted dying following a negative advice. Furthermore, specialization trainings and health services have been purposely developed in various jurisdictions to support and educate consultants in assisted dying practice and assisted dying request assessments: for instance, “Canadian Association of MAiD Assessors and Providers” (CAMAP) in Canada, “Voluntary Assisted Dying Medical Practitioner Training” in Western Australia, “Support and Consultation in Euthanasia Networks” (SCEN) in the Netherlands, “Support and Consultation for End of Life in New Zealand” (SCENZ) Group, and “Life End Information Forum” (LEIF) in Flanders and Brussels (Belgium).

Notwithstanding the acknowledged importance of consulting trained assisted dying consultants (8), empirical evidence on this practice is rather limited. Furthermore, previous research has especially studied the practice from the consultants' accounts, and less from the attending practitioners' perspectives (9, 10). This might have led to some bias. In addition, previous research has been mainly conducted in the early-adopting jurisdictions (such as Belgium and the Netherlands) in the early years after implementing assisted dying legislation (11–13). Therefore, the peer consultation practice can be assumed to have changed over time, as assisted dying practice has undergone some shifts as well (14–17). Therefore, studying the current peer consultation practice, and more specifically its peer consultations, can provide various important insights. Firstly, it can indicate which attending physicians seek the support of trained assisted dying consultants and for which cases, thus revealing the support needs of attending physicians in exploring an assisted dying request. Secondly, it can shed light on how trained assisted dying consultants assess the cases for which consultation has been sought, thereby exploring the relationship between their advices and the cases. In other words, insights can be used to identify routes to improve the care and support for patients requesting assisted dying, as well as for attending physicians who consult trained consultants.

Therefore, the aim of our study was to investigate the characteristics and outcomes of peer consultations for assisted dying request assessments between attending physicians and trained assisted dying consultants, as reported by the attending physicians. More specifically, we examined the peer consultation practice with trained assisted dying consultants in Flanders and Brussels (Belgium)—i.e., Life End Information Forum (LEIF) consultants—from the perspectives of attending physicians. The research questions are the following:

1. What are the characteristics of attending physicians who hold peer consultations for assisted dying request assessments with trained assisted dying (LEIF) consultants?
2. What are the characteristics of persons requesting assisted dying and of their requests in the peer consultations for assisted dying request assessments with trained assisted dying (LEIF) consultants?
3. What are the characteristics of the peer consultations for assisted dying request assessments with trained assisted dying (LEIF) consultants?
4. What are the outcomes of the peer consultations for assisted dying request assessments with trained assisted dying (LEIF) consultants in terms of consultants' advices on substantive requirements and in terms of assisted dying being performed?
5. Which characteristics of persons requesting assisted dying, characteristics of the requests, peer consultation characteristics, and consultants' advices are associated with the performance of assisted dying?

## 2. Methods

### 2.1. Study design, setting and participants

We conducted a cross-sectional survey study among attending physicians who assessed an assisted dying request in the year prior to the study and who held a peer consultation with a LEIF consultant as legally mandatory second or third physician. A LEIF consultant is a physician who has followed the “LEIF Physician Training”. This training consists of five modules, each lasting 5.5 h: (1) medical end-of-life decisions, the Belgian assisted dying legislation, and the Belgian legislation on patient rights and access to palliative care, (2) the organization and functioning of LEIF, the legal context for advance directives and advance care planning, (3) ethics and the concept of mental capacity in palliative care, (4) assisted dying in practice and research, and (5) physician communication with patients, relatives of patients, and other professional caregivers in the context of end-of-life decisions (18). LEIF consultants perform peer consultations for assisted dying request assessments only in the Dutch-speaking region of Belgium: namely, the Brussels Capital Region and Flanders. Thus, this study was carried out in a region that comprises 68% of the Belgian population. We followed the STROBE guidelines in reporting this cross-sectional study (19).

To identify eligible participants, we used the database of the LEIF organization, in which the peer consultations and the physicians involved are registered for reimbursement from the Belgian National Institute for Health and Disability Insurance (NIHDI). Registration of peer consultation is not mandatory. A peer consultation is only registered when the LEIF consultant seeks reimbursement for the consultation performed. Furthermore, only those LEIF consultants who are licensed in advance by the NIHDI can request such reimbursement. In total, we identified 904 attending physicians as eligible for study inclusion. Eligibility was defined as having consulted a LEIF consultant in the year prior to the study. In 2019, LEIF consultants and End of Life consultants (i.e., Walloon counterparts of LEIF consultants) acted as second or third physician in 27% of the 2655 assisted dying cases reported to the FCECE (20).

### 2.2. Data collection

From September 2019 to May 2020, we sent pen and paper questionnaires to the work addresses of the attending physicians following Dillman's Total Design Method (21). This included participants receiving up to three reminders for study participation when no response was received. A duplicate of the questionnaire was included in the second reminder. Participants could answer the questionnaire either on paper (returning it in the prepaid envelope included) or online through a website developed using Limesurvey. Each participant was assigned a unique ID code to enable follow-up of responses and to ensure the participant's anonymity.

### 2.3. Questionnaire and main measure instruments

We used a 4-page pre-structured questionnaire similar to the one used in a previous study by Van Wesemael and colleagues conducted in 2008 (22). Minor modifications to the original questionnaire were made to adapt it to the current context of assisted dying practice. Our questionnaire included questions about (1) the attending physician's socio-demographic characteristics and experience with palliative and end-of-life care, (2) the characteristics of his or her most recent peer consultation for an assisted dying request assessment with a LEIF consultant in the 12 months prior to the study, and (3) his or her attitudes toward consulting a LEIF consultant for an assisted dying request assessment. To measure outcomes of assisted dying, close-ended questions were included on the LEIF consultant's advice of the peer consultation and whether or not assisted dying had been performed following the peer consultation.

With regard to the advice from the consultant—i.e., the outcome of the assisted dying request—the answer options consisted of: (1) The LEIF consultant gave the positive advice in that substantive requirements were met, (2) The LEIF consultant gave the negative advice in that substantive requirements were not met, and (3) The LEIF consultant did not give advice. With regard to whether assisted dying had been performed, the answer options consisted of: (1) Yes, I carried out the assisted dying, (2) No, I rejected the assisted dying request, (3) No, the patient had withdrawn the request, (4) No, the patient had died before the performance, (5) Yes, the LEIF consultant carried out the assisted dying, and (6) Yes, another physician carried out the assisted dying.

### 2.4. Ethical considerations

This study and its study materials were approved by the Medical Ethics Committee of the University Hospital of Brussels (B.U.N. 143201939962; March 24, 2019). The participants received information about the aim and the design of the study in a cover letter. For the postal questionnaire, informed consent was assumed upon return. For the online questionnaire, informed consent was explicitly requested.

### 2.5. Statistical analysis

To answer the first, second, third, and fourth research questions, we performed descriptive analyses. Descriptive summaries are presented as N (%) and percentages were rounded up. To answer the fifth research question, we performed univariable logistic regression analyses. The dependent variable “assisted dying being performed” is based on the survey question “Did you carry out the assisted dying following the peer consultation?” We dichotomized answer options to this question into “assisted dying not being performed” (“No, I rejected the assisted dying request”; “No, the patient had withdrawn the request”; and “No, the patient had died before the performance”) and “assisted dying being performed” (“Yes, I carried out the assisted dying”; “No, the LEIF consultant carried out the assisted dying”; and “Yes, another physician carried out the assisted dying”). Univariable odds ratios with 95% confidence intervals were calculated for: the characteristics of persons requesting assisted dying and their requests, for peer consultation characteristics (interrelationship characteristics, reason(s) for consulting a LEIF consultant, and the attending physician's attitude toward the request prior to consultation), and for LEIF consultants' advices on substantive requirements. An alpha level of p < 0.05 defined statistical significance. Missing data were removed from analysis (listwise). We did not apply correction for multiple testing because of the exploratory nature of this study, to avoid missing out on potentially valuable results that initially appear not significant but have research potential for future confirmatory studies (23). Statistical analyses were performed using SPSS IBM 27.

## 3. Results

We received a response from 503 attending physicians. We excluded one physician from the study sample, as he or she had not consulted a LEIF consultant to assess an assisted dying request in the 12 months prior to the study. This results in a valid response rate of 56% (502/903).

### 3.1. Characteristics of attending physicians

The attending physicians were mainly men (58.8%) and general practitioners (91.6%) (Table 1). The majority of the attending physicians (57.8%) were 50 years old or older. More than three-quarters of the attending physicians had followed an additional end-of-life or palliative care training (75.9%). More specifically, about one out of ten attending physicians had completed a postgraduate interuniversity training in palliative care (12.9%) or the Life End Information Forum (LEIF) physician training (10.6%), while a small group (5.0%) had followed training in palliative care for patients with incurable illness. Nearly half of the attending physicians (46.2%) had attended study days and seminars about pain management, and two out of five (38.8%) about advance care planning. Two out of five attending physicians (42.5%) had cared for fewer than five incurably ill patients at the end of life in the year prior to the survey. The majority of attending physicians (87.2%) had already consulted a second or third physician for assessing another assisted dying request prior to the most recent assessment.

**Table 1:** Characteristics of attending physicians who held peer consultations for assisted dying request assessments with trained assisted dying (LEIF) consultants (*N* = 502).

	***N*** **(%)**
**Sex, Male**	263 (58.8)
**Age**	
<40 years	109 (21.8)
40–49 years	102 (20.4)
50–59 years	125 (25.0)
≥60 years	164 (32.8)
**Medical specialty**	
General practitioner	456 (91.6)
Other medical specialist	42 (8.4)
**Additional end-of-life/palliative care training** ^*^ **:**	381 (75.9)
Postgraduate interuniversity training in palliative care	64 (12.9)
Life End Information Forum (LEIF) physician training^**^	53 (10.6)
Training in palliative care for patients with incurable illness^§^	25 (5.0)
Study days and seminars about:	
Pain management	230 (46.2)
Advance care planning	193 (38.8)
Breaking bad news	90 (18.1)
Bereavement counseling	65 (13.1)
Existential and spiritual care	36 (7.2)
**Incurably ill patients at the end of life cared for in the year prior to the survey**	
<5 patients	204 (42.5)
5–9 patients	150 (31.3)
10–19 patients	95 (19.8)
≥20 patients	31 (6.5)
**Consulted a second or third physician to assess another assisted dying request prior to most recent peer consultation**	431 (87.2)

Percentages may not always add to 100% because of rounding.

Missing values: Sex: *n* = 55, Age: *n* = 2; Medical specialty: *n* = 4; Additional end-of-life/palliative care training: *n* = 4; Incurably ill patients at the end of life: *n* = 22; Consulted a 2nd or 3rd physician: *n* = 8.

LEIF: Life End Information Forum.

* Multiple answers were possible.

** Five-day training focusing on assisted dying, other medical practices at the end of life, and quality criteria for consultation in assessing assisted dying requests as attending physician or consultant.

§ Five-day training focusing on palliative care for people with incurable illness.

### 3.2. Characteristics of persons requesting assisted dying and of their requests

In about half of consultations (55.5%), the patient requesting assisted dying was female. In more than half of consultations (66.1%), the patient was at least 70 years old, and the main diagnosis was cancer (56.5%) (Table 2). General deterioration was the main diagnosis in 14.8% of the consultations, and neurological disorder in 7.5%. In 4.9% of consultations, psychiatric disorder was the main diagnosis. Suffering without prospect of improvement was indicated as one of the patient's reasons for requesting assisted dying in 80.7% of consultations, general weakness or tiredness in 45%, and loss of dignity in 39.4%. In about three out of ten consultations, loss of independence (30.7%), pain (28.7%), tiredness of life (27.9%), or not wanting to be a burden on the family/environment (26.3%) was reported as one of the patient's reasons for requesting assisted dying. When it comes to the patient's most important reason for requesting assisted dying, suffering without prospect of improvement was indicated in nearly half of the consultations (49.3%). In about one out of ten consultations, loss of dignity (11.3%), pain (9.0%), or tiredness of life (9.0%) was reported as the patient's most important reason for requesting assisted dying.

**Table 2:** Characteristics of persons requesting assisted dying (patients) and of their requests (*N* = 502).

	***N*** **(%)**
**Characteristics of patients**	
**Sex, Male**	214 (44.5)
**Age**	
<60 years	65 (13.3)
60–69 years	100 (20.5)
70–79 years	130 (26.7)
≥80 years	192 (39.4)
**Main diagnosis**	
Cancer	278 (56.5)
General deterioration	73 (14.8)
Neurological disorder^*^	37 (7.5)
Psychiatric disorder	24 (4.9)
Heart failure	14 (2.8)
Chronic obstructive pulmonary disease (COPD)	14 (2.8)
Other diagnosis^§^	52 (10.3)
**Characteristics of requests reason(s) for requesting assisted dying** ^**^	
Suffering without prospect of improvement	402 (80.7)
General weakness or tiredness	224 (45.0)
Loss of dignity	196 (39.4)
Loss of independence in daily life	153 (30.7)
Pain	143 (28.7)
Tiredness of life	139 (27.9)
Not wanting to be a burden on the family or environment	131 (26.3)
Disability	92 (18.5)
Depression	38 (7.6)
Anxiety	33 (6.6)
Fear of suffocation	27 (5.4)
Vomiting	14 (2.8)
Other reason(s)‡	24 (4.8)
**Most important reason for requesting assisted dying**	
Suffering without prospect of improvement	236 (49.3)
Loss of dignity	54 (11.3)
Pain	43 (9.0)
Tiredness of life	43 (9.0)
Not wanting to be a burden on the family or environment	27 (5.6)
General weakness or tiredness	23 (4.8)
Loss of independence in daily living	13 (2.7)
Disability	10 (2.1)
Anxiety	10 (2.1)
Fear of suffocation	8 (1.7)
Depression	4 (0.8)
Vomiting	0 (0.0)
Other reason	8 (1.7)
**Patient made a written request**	464 (95.9)

Percentages may not always add to 100% because of rounding.

Missing values: Sex: *n* = 21; Age: *n* = 15; Main diagnosis: *n* = 11; Most important reason for requesting assisted dying: *n* = 24; Patient made a written request: *n* = 19.

LEIF, Life End Information Forum.

* Neurological disorder includes “MS/ALS”, “early stage of dementia”, and “cerebrovascular accident”.

§ Examples include “dyskeratosis congenital”, “systemic scleroderma”, “gangrene”, “cervical spinal stenosis”, “interstitial lung disease”, “chronic kidney disease (CKD)”, and “primary biliary cholangitis”.

** Multiple answers were possible.

^‡^Examples include “fear of losing control of life”, “blindness”, and “loss of quality of life”.

### 3.3. Peer consultation characteristics

The attending physician knew the LEIF consultant in about nine out of ten consultations (87.7%), most frequently as a practitioner in the same region (55.9%) (Table 3). In a small proportion of consultations (4.1%), the LEIF consultant knew the patient. In the vast majority of consultations (85.1%), the attending physician consulted the LEIF consultant because of his or her expertise, and in nearly half of the consultations (46.1%) because of his or her independence as second physician. In 78.6% of the consultations, the attending physician had decided to grant the request prior to the consultation, in 19.1% he or she had not made a final decision yet, and in 2.3% he or she had already decided not to grant the request. In the majority of consultations, the attending physician discussed the medical hopelessness of the case (83.9%), the unbearable nature of the suffering (70.7%), the well-considered nature of the request (69.3%), the voluntariness of the request (56.6%), or the durability of the request (55.2%). In about three out ten consultations (27.5%), the attending physician asked questions about the practical performance of assisted dying. In one-quarter of the consultations (25.9%), the attending physician requested the LEIF consultant to assist with the performance of assisted dying. In 15.3% of the consultations, the attending physician requested the LEIF consultant to carry out the assisted dying (i.e., to administer the lethal drugs).

**Table 3:** Characteristics of peer consultations for assisted dying request assessments with trained assisted dying (LEIF) consultants (*N* = 502).

	***N*** **(%)**
**Interrelationship characteristics**	
**Physician-consultant relationship**	
Attending physician did not know the LEIF consultant	61 (12.3)
Attending physician knew the LEIF consultant^*^:	436 (87.7)
As a practitioner in the same region	278 (55.9)
Because he/she had already consulted the LEIF consultant before	124 (24.9)
As a befriended colleague	75 (15.1)
Known only by name	67 (13.5)
As a colleague from the same hospital	23 (4.6)
As a colleague from the same practice	14 (2.8)
In another way^**^	10 (2.1)
**Patient-consultant relationship**	
LEIF consultant did not know the patient	466 (95.9)
LEIF consultant knew the patient as co-treating practitioner	7 (1.4)
LEIF consultant knew the patient in another way	13 (2.7)
**Process characteristics**	
**Reasons for consulting a trained assisted dying (LEIF) consultant** ^*^	
Expertise of the LEIF consultant as second physician	423 (85.1)
Independence of the LEIF consultant as second physician	229 (46.1)
Questions about the legal procedure	158 (31.8)
Questions about the practical performance of assisted dying	137 (27.6)
Accessibility to/availability of LEIF consultants	129 (26.0)
Complexity of the assisted dying request	120 (24.1)
To assess the own evaluation of the assisted dying request	115 (23.1)
To avoid burdening colleagues	30 (6.0)
No other second physician was known or available	18 (3.6)
Other reason(s)†	27 (5.4)
**Attending physician's attitude toward the request prior to the peer consultation**	
Had decided to grant the request	383 (78.6)
Had not made a final decision yet regarding the request	93 (19.1)
Had decided to not grant the request	11 (2.3)
**Topics discussed during the peer consultation** ^*^	
Medical hopelessness of the case	418 (83.9)
Unbearable nature of the patient's suffering	352 (70.7)
Well-considered nature of the request	345 (69.3)
Voluntariness of the request	282 (56.6)
Durability of the request	275 (55.2)
Expected time frame until death	219 (44.0)
Moment of performing assisted dying	167 (33.5)
Whether it was justified to perform assisted dying in the particular case	135 (27.1)
Whether the LEIF consultant would assist in performing assisted dying	129 (25.9)
Method of carrying out assisted dying, e.g., which drugs to use	128 (25.7)
Possible alternative palliative treatments	114 (22.9)
The place where assisted dying would be carried out	103 (20.7)
The registration with the Federal Control and Evaluation Commission for Euthanasia (FCECE)	95 (19.1)
Whether the LEIF consultant would be willing to carry out the assisted dying	76 (15.3)
Possible alternative curative treatments	64 (12.9)
Legal aspects	40 (8.0)
Oher topic(s)^¶^	10 (2.0)

Percentages may not always add to 100% because of rounding.

Missing values: Attending physician-consultant relationship: *n* = 5; Patient-consultant relationship: *n* = 16; Reasons for consulting a LEIF consultant: *n* = 5; Attending physician's attitude toward the request prior to consulting: *n* = 16; Topics discussed during the peer consultation with the LEIF consultant: *n* = 5.

LEIF, Life End Information Forum.

* Multiple answers were possible.

** Examples include “as former co-student”, “as a colleague from same palliative network”, and “as a former lecturer”.

^†^Examples include “difficulties with the assisted dying procedure”, “LEIF consultant was a colleague”, and “the pressing nature of the patient's medical condition”.

¶ Examples include “technical difficulties encountered in previous performances of assisted dying”, “funeral arrangement”, and “how to explain the eligibility of the request to the family”.

### 3.4. Peer advices on substantive requirements and outcomes of the assisted dying requests

**Figure 1:**
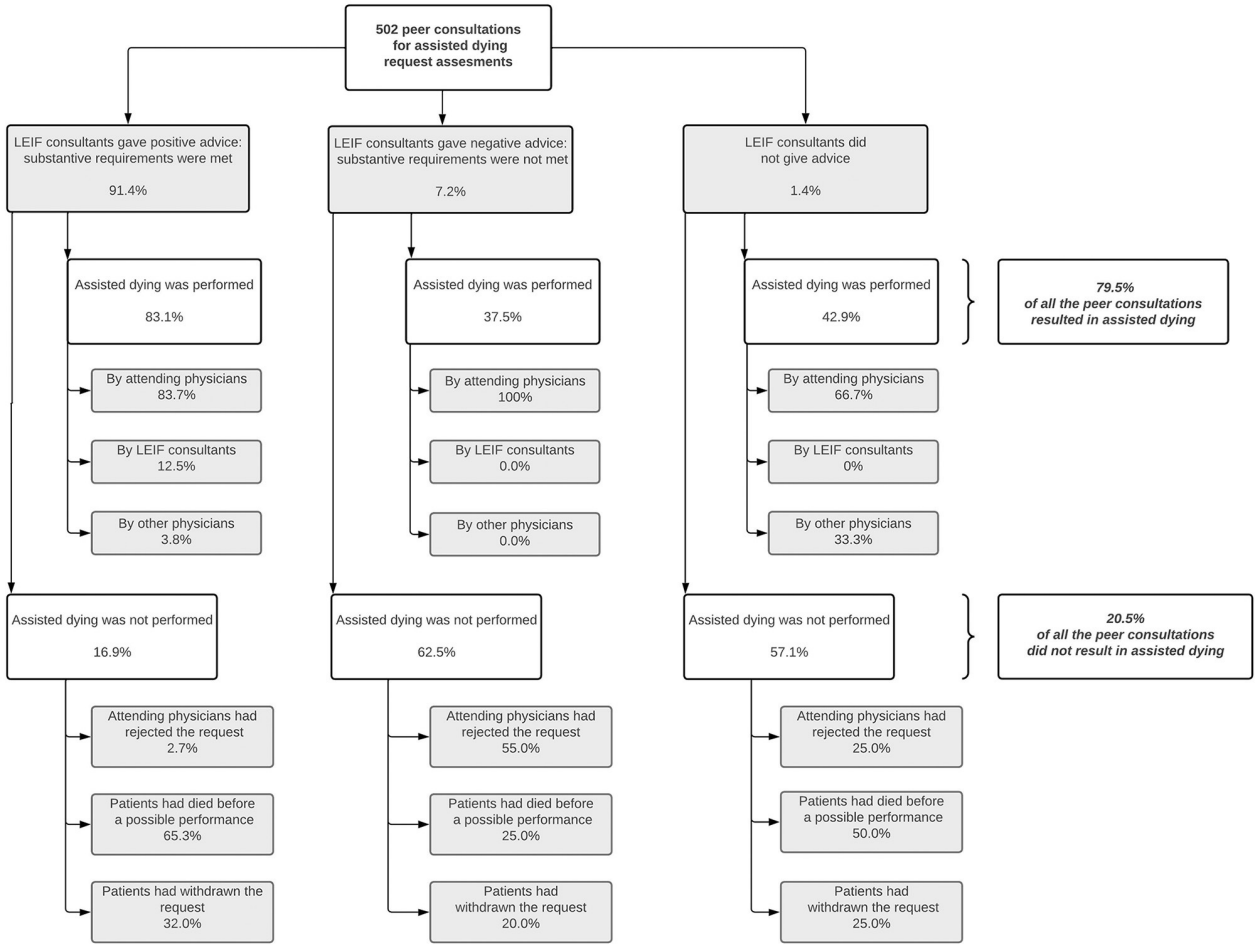
Advices from life end information forum (LEIF) consultants on whether substantive requirements were met in the most recent peer consultations for an assisted dying request assessment, and the outcomes of these assisted dying requests following the consultations (within-group percentages). Missing values range from 3.0% to 3.8%. Percentages may not always add to 100% because of rounding.

The LEIF consultant gave a positive advice—i.e., substantive requirements were met—in 91.4% of the consultations, and a negative advice – i.e., substantive requirements were not met—in 7.2% (Figure 1). In 1.4% of the consultations, the LEIF consultant did not give an advice. Four out of five consultations resulted in the performance of assisted dying (79.5%). Of all the performances of assisted dying, 83.7% were carried out by the attending physician, 12.5% by the LEIF consultant, and 3.8% by another physician. One out of five consultations resulted in assisted dying not being performed (20.5%). For all cases in which assisted dying was not performed: 14.1% were because the attending physicians had rejected the request, in 56.6% the patient had died before the possible performance of assisted dying, and in 29.3% the patient had withdrawn the request.

### 3.5. Characteristics of persons requesting assisted dying, characteristics of the requests, peer consultation characteristics, and peer advices associated with the performance of assisted dying

Consultations for patients with a psychiatric disorder were less likely to result in the performance of assisted dying compared to consultations for patients with cancer (47.8% vs. 81.2%, OR 0.21, 95% CI: 0.09–0.51) (Table 4). Consultations in which loss of dignity (85.2% vs. 75.6%, OR 1.86, 95% CI: 1.15–2.99), general weakness or tiredness (85.2% vs. 74.6%, OR 1.96, 95% CI: 1.23–3.12), or loss of independence in daily living (85.4% vs. 76.4%, OR 1.77, 95% CI: 1.06–2.98) was reported as one of the patient's reasons for requesting assisted dying were more likely to result in the performance of assisted dying, compared to consultations in which one of these reasons was not reported. Consultations in which depression was indicated as one of the patient's reasons for requesting assisted dying had lower odds of resulting in the performance of assisted dying (59.5% vs. 81.1%, OR 0.34, 95% CI: 0.17–0.69). Consultations in which not wanting to be a burden on the family or environment (63.0% vs. 82.0%, OR 0.37, 95% CI: 0.16–0.87) or fear of suffocation (50.0% vs. 82.0%, OR 0.22, 95% CI: 0.05–0.92) was indicated as the patient's most important reason for requesting assisted dying were less likely to lead to the performance of assisted dying, compared to consultations in which suffering without prospect of improvement was indicated as the patient's most important reason. Consultations that included questions about the practical performance of assisted dying were more likely to result in the performance of assisted dying, compared to consultations that did not include these questions (86.9% vs. 76.6%, OR 2.02, 95% CI: 1.16–3.52). Consultations in which the complexity of the assisted dying request was discussed were less likely to result in the performance of assisted dying, compared to consultations in which this was not discussed (68.4% vs. 83.0%, OR 0.44, 95% CI: 0.28–0.71%). Consultations in which the attending physician had decided to grant the request prior to consultation were more likely to result in the performance of assisted dying, compared to consultations in which the attending physician had decided not to grant the request prior to consultation (84.6% vs. 60.0%, OR 4.56, 95% CI: 1.35–15.44). Furthermore, consultations in which the LEIF consultant gave a positive advice (i.e., substantive requirements were met) were more likely to result in the performance of assisted dying, compared to consultations in which the LEIF consultant gave a negative advice (i.e., substantive requirements were not met) (83.1% vs. 37.5% OR 8.20, 95% CI: 3.84–17.49).

**Table 4:** Characteristics of persons requesting assisted dying (patients), characteristics of requests, peer consultation characteristics, and peer advices on substantive requirements associated with the performance of assisted dying (*N* = 502).

	**Assisted dying**	
	**Row %**	**Univariable odds ratio (95% CI)**
**Characteristics of patients and requests**		
**Sex**		
Male	80.1	Reference
Female	78.8	0.87 (0.55–1.36)
**Age**		
<60 years	80.3	Reference
60–69 years	78.0	0.87 (0.39–1.91)
70–79 years	78.5	0.89 (0.42–1.90)
≥80 years	80.7	1.03 (0.50–2.12)
**Main diagnosis**		
Cancer	81.2	Reference
General deterioration	82.2	1.07 (0.55–2.10)
Neurological disorder*	80.6	0.96 (0.40–2.32)
Psychiatric disorder	47.8	**0.21 (0.09–0.51)**
Heart failure	76.9	0.77 (0.21–2.91)
Chronic obstructive pulmonary disease (COPD)	85.7	1.39 (0.30–6.43)
Other diagnosis^§^	78.0	0.82 (0.40–1.72)
**Reason(s) for requesting assisted dying** ^**^		
Suffering without prospect of improvement	80.5	1.37 (0.79–2.36)
Loss of dignity	85.2	**1.86 (1.15–2.99)**
Pain	81.1	1.16 (0.71–1.89)
Tiredness of life	79.0	0.96 (0.59–1.56)
Not wanting to be a burden on the family/environment	78.3	0.91 (0.56–1.49)
General weakness or tiredness	85.2	**1.96 (1.23–3.11)**
Loss of independence in daily life	85.4	**1.77 (1.06–2.98)**
Disability	84.6	1.53 (0.82–2.83)
Anxiety	81.8	1.18 (0.47–2.93)
Depression	59.5	**0.34 (0.17–0.69)**
Fear of suffocation	81.5	1.15 (0.42–3.10)
Vomiting	85.7	1.57 (0.35–7.12)
Other reason(s)	83.3	1.31 (0.44–3.92)
**Most important reason for requesting assisted dying**		
Suffering without prospect of improvement	82.0	Reference
Loss of dignity	90.7	2.16 (0.81–5.74)
Pain	76.7	0.73 (0.33–1.59)
Tiredness of life	72.1	0.59 (0.27–1.20)
Not wanting to be a burden on the family/environment	63.0	**0.37 (0.16–0.87)**
General weakness or tiredness	82.6	1.05 (0.34–3.23)
Loss of independence in daily living	75.0	0.66 (0.17–2.54)
Disability	80.0	0.88 (0.18–4.29)
Anxiety	80.0	0.88 (0.18–4.29)
Depression	33.3	0.11 (0.01–1.24)
Fear of suffocation	50.0	**0.22 (0.05–0.92)**
Other reason	87.5	1.54 (0.18–12.85)
**Peer consultation characteristics**		
**Attending physician-consultant relationship**		
Consultant did not know the attending physician	77.6	Reference
Consultant knew the attending physician	79.7	0.71 (0.59–2.20)
**Patient-consultant relationship**		
LEIF consultant did not know the patient	79.2	Reference
LEIF consultant knew the patient‡	84.2	0.72 (0.20–2.50)
**Reason(s) for consulting a trained assisted dying (LEIF) consultant** ^**^		
Expertise of the LEIF consultant as second physician	79.7	1.10 (0.60–2.07)
Independence of the LEIF consultant as a second physician	81.9	1.33 (0.85–2.08)
Questions about the legal procedure	81.5	1.21 (0.75–1.96)
Questions about the practical performance of assisted dying	86.9	**2.02 (1.16–3.52)**
Accessibility to/availability of the LEIF consultant	83.6	1.44 (0.85–2.44)
Complexity of the assisted dying request	68.4	**0.44 (0.28–0.71)**
To assess the own evaluation of the assisted dying request	77.2	0.84 (0.51–1.39)
To avoid the burdening of colleagues	79.3	0.99 (0.39–2.50)
No other second physician was known or available	88.9	2.11 (0.48–9.35)
Other reason(s)	85.2	1.52 (0.51–4.49)
**Attending physician's attitude toward the request prior to the peer consultation**		
Had decided not to grant the request	60.0	Reference
Had decided to grant the request	84.6	**4.56 (1.35**–**15.44)**
Had not made a final decision yet	54.5	1.25 (0.36–4.41)
**Assisted dying (LEIF) consultant's advice on substantive requirements**		
Negative advice: substantive requirements were not met	37.5	Reference
Positive advice: substantive requirements were met	83.1	**8.20 (3.84**–**17.49)**
Did not gave advice	42.9	1.25 (0.24–6.57)

Missing values range from 3.0 to 5.6%.

Percentages are row percentages.

Bold indicates statistical significance (*p* < 0.05).

CF, confidence interval; LEIF, Life End Information Forum.

^*^Neurological disorder includes “MS/ALS”, “early stage of dementia”, and “cerebrovascular accident”.

** Multiple answers were possible. Answer option “no” is the reference category.

^‡^LEIF consultant knew the patient either as co-treating practitioner or in another way.

## 4. Discussion

### 4.1. Main findings

The large majority of attending physicians consulting a trained assisted dying (LEIF) consultant for an assisted dying request assessment were general practitioners. The majority of peer consultations concerned patients with cancer, and a considerable proportion concerned patients with general deterioration. In nine out of ten peer consultations, LEIF consultants gave a positive advice—i.e., substantive requirements were met. About four out of five peer consultations resulted in the performance of assisted dying. Peer consultations in which loss of dignity, loss of independence in daily living, or general weakness or tiredness was reported as the patient's reason for requesting assisted dying were more likely to result in the performance of assisted dying. Peer consultations in which psychiatric disorder was reported as diagnosis were less likely to result in the performance of assisted dying.

### 4.2. Strengths and limitations

Our study has several strengths. First of all, it focused on describing the characteristics of peer consultations for assessing assisted dying requests, whereas other studies have mainly focused on those of the actual performance of assisted dying. This topic has been scarcely addressed in recent literature. Hence, our findings may be particularly relevant for the vast majority of jurisdictions with assisted dying legislation where peer consultation is legally required as well, such as in Canada, Spain, the Netherlands, several U.S. states, New Zealand, several Australian states, and Luxembourg. Secondly, we obtained a relatively high response rate for a physician survey study. Most likely, this stems from the robust mailing procedure and the questionnaire being available both online and on paper to reduce technical barriers. Thirdly, we only collected data on the most recent peer consultations to reduce recall bias. With regard to limitations, recall bias may be possible, especially for data on peer consultations that were carried out several months prior to the survey. Moreover, it is possible that there was some ascertainment bias, as only those peer consultations were included for which LEIF consultants requested a reimbursement from the NIHDI. Moreover, some selection bias might have occurred, as we might have obtained a higher study participation by those physicians who have a particular interest in assisted dying or who endorse the importance of peer consultation in assisted dying.

### 4.3. Interpretation of findings

Our findings suggest that the consultation practice with LEIF consultants is most often embedded in a specific setting—namely, in a primary care setting where both attending physicians and consultants are acquainted with each other. First of all, this is substantiated by the majority of attending physicians (92%) in our study who were general practitioners. This corroborates previous research on assisted dying request assessments by trained assisted dying consultants in Belgium and the Netherlands (10, 11, 22, 24, 25). In a previous study, we also found that the majority of LEIF consultants (72%) were general practitioners (18). In contrast, other studies have suggested that about 40%–60% of assisted dying cases in Belgium are carried out by general practitioners (26, 27). Consequently, it appears that attending physicians with a medical specialty other than general medicine consult non-LEIF consultants, thereby suggesting an alternative ‘circuit' of mandatory peer consultations in non-primary care settings such as hospitals. Probably, medical specialists in these settings have easier and adequate access to relevant expertise or peers to assess an assisted dying request. Future research could examine that specific peer consultation practice and whether its characteristics differ from the LEIF practice. Secondly, the specific setting of the LEIF practice is also substantiated by a high proportion of attending physicians who indicated knowing the consultant in some manner, mostly as a practitioner in the same region. Moreover, some attending physicians reported that the LEIF consultant was a befriended colleague. This could imply that they put emphasis on having a trust relationship with consultants in assisted dying practice, which can facilitate open communication and is related to better healthcare delivery and outcomes for patients (28). Although this finding does not allow us to make sound conclusions about the legally required independence between attending physicians and consultants, it may prompt further conceptual reflection on its inherent meaning as it has not been specifically defined in Belgian assisted dying legislation. However, the FCECE interprets independence as the fact that the attending physician cannot have family or hierarchical ties with the consultant (6).

Our study presents a broader picture of the context of peer consultations with trained assisted dying consultants by both examining attending physicians' reasons to initiate them and their outcomes. With regard to the latter, we found that the LEIF consultant gave a positive advice (i.e., the patient was eligible for assisted dying) in the vast majority of peer consultations (91%). This is more than reported by studies in the Netherlands, in which the figure is four out of five (10). Moreover, those peer consultations with a positive advice were considerably more likely to result in assisted dying compared to consultations with a negative advice, despite the advice not being binding. The large proportion (88%) of cases in which the consultant knew the attending physician would seem a plausible reason for the large proportion (91%) of cases receiving a positive advice. However, such a conclusion is not warranted by the data following the fact that cases in which the consultant did not know the attending physician were not less likely to result in assisted dying compared to those in which the consultant did know the attending physician. Notwithstanding, future research could confirm that hypothesis by investigating what characteristics of peer consultations influence a positive advice. On the other hand, the large proportion of positive advices may indicate that attending physicians approach assisted dying requests with considerable due care, only contacting LEIF consultants when there is a high chance of the patient being eligible for assisted dying. This could explain our finding that more than three-quarters (79%) of attending physicians had already decided to grant the request prior to the advice. That result may also suggest that attending physicians indeed view these peer consultations as means of validating their own decision concerning the patient's eligibility, as intended by assisted dying legislation in Belgium. However, one may question the added value of peer consulting when the attending physician has already made a decision beforehand: in such cases, is the peer consultation merely a ‘tick the box exercise'—i.e., merely meeting the procedural requirement to be legally compliant? Or more generally, what is the added value of peer consultation, as attending physicians are not legally obliged to adhere to the peer advice? Our findings provide more nuance to such inquiries, showing that attending physicians also approach peer consultation as an opportunity to fulfill their specific support needs regarding assisted dying. These support needs are reflected in their reasons to hold peer consultations with trained assisted dying consultants. For example, some attending physicians consulted LEIF consultants for medical-technical questions about the performance (28%), requesting assistance in the actual performance (26%), or requesting consultants to carry out the actual performance of assisted dying (15%). In fact, some LEIF consultants carried out the assisted dying following the peer consultations. Engaging trained assisted dying consultants in the performance of assisted dying might be good medical practice. Their specific expertise can be useful when attending physicians experience difficulties or challenges during the performance (for instance, finding the proper vein for injecting the lethal drugs). However, some questions can be raised as well about consultants carrying out assisted dying. Had they become the new attending physicians of the patients concerned? In this case, another independent consultant must have been consulted again in order to assess the request. Alternatively, it could be that the consultant was present during the performance and took over at the very last moment (for example, because the attending physician was ultimately not capable of performing it)? More insights into the specific context of this phenomenon are warranted. Regardless of consultants' motives for administering the lethal drugs instead of the attending physicians, the physicians involved should consider to what extent this is in line with assisted dying legislation in Belgium. Furthermore, we found that certain legal requirements were not explicitly discussed in some peer consultations: 84% discussed the medical hopelessness of the case, 71% the unbearable nature of the patient's suffering, 69% the well-considered nature of the request, 57% the voluntariness of the request, and 55% the durability of the request. Discussing all legal requirements can be viewed as a quality criterion for peer consultation in assisted dying practice (11, 18).

Lastly, our study provides a novel characterization of the patient population requesting and receiving assisted dying. Firstly, we found that persons with cancer were the largest patient group requesting assisted dying (57%). This confirms previous research (16, 27, 29, 30). However, the proportion of patients with cancer in our study is notably lower compared to similar studies among trained assisted dying consultants in Belgium in 2009 and in the Netherlands in 2011 (10, 22). In these past studies, patients with cancer represented three-quarters of the consultations. This change might indicate that the assessments concerning patients with cancer are commonly perceived as less complex because of the predictability of the disease trajectory, and, as a result, attending physicians may feel less need to rely on the expertise of trained assisted dying consultants, and therefore consult physicians without special assisted dying training (9). Secondly, the proportion of peer consultations concerning patients with general deterioration has increased compared to previous similar studies: from 7% then to 15% now (22). Furthermore, cases reporting general weakness or loss of dignity as a reason for requesting assisted dying were more likely to result in the performance of assisted dying, compared to peer consultations in which these reasons were not reported. That is in line with evidence from the Netherlands (10, 31), and our findings seem to confirm the trend that persons with old-age-related conditions are currently more often requesting and receiving assisted dying than in the early years of legislation (15, 18, 32). This could be attributed to attending physicians being increasingly open to proceeding with such requests. However, these assessments are commonly perceived as less clear-cut and more challenging in comparison with those from patients with cancer (9, 33). Furthermore, attending physicians may feel better supported by trained assisted dying consultants, and may consult them more frequently for such cases. Thirdly, 5% of peer consultations concerned patients with psychiatric conditions and were less likely to result in the performance of assisted dying compared to those concerning patients with cancer. This is in line with Dutch studies (24, 34), and might indicate that attending physicians are willing to explore these patients' eligibility for assisted dying but are rather reluctant to carry it out afterwards (35). It could also be that they were less willing to perform assisted dying in these patients due to a highly mediatized prosecution of physicians involved in the assisted dying case of a patient with a psychiatric condition in Flanders (Belgium) in 2020 (36). Alternatively, attending physicians may have refused to perform assisted dying due to a negative peer advice (i.e., not all substantive requirement were fulfilled—for example, because not all reasonable therapeutic options had been utilized) (37, 38). Another explanation could be that assisted dying was not performed because patients with psychiatric conditions may tend to withdraw their request or put it on hold (39, 40). Thus, our findings suggest that attending physicians may consider reasons related to psychological dimensions of suffering—e.g., loss of dignity, general weakness, and loss of independence—as compelling for granting assisted dying, but they may approach ‘psychological reasons' differently in patients with psychiatric conditions. In other words, differences in medical diagnosis between patient groups might explain differences in receiving assisted dying.

## 5. Conclusion

Examining peer consultations for assisted dying request assessments provides important insights into assisted dying practices, as it sheds light on the dynamics prior to performance. Our findings indicate that the peer consultation practice with trained assisted dying consultants is most often embedded in a primary care setting. Moreover, our study corroborates previous research in that an increasing proportion of assisted dying consultations concerns patients with general deterioration, whereas in earlier periods after the implementation of the assisted dying law this most often concerned patients with cancer. Attending physicians seem to hold peer consultations to validate their own decision-making and to request additional support, especially in relation to the actual performance. Therefore, support in assisted dying should be aligned with the challenges of current practice, while paying particular attention to the preparation for, and the act of, performance in order to adequately meet the needs of attending physicians.

## Data availability statement

The datasets presented in this article are not readily available because of GDPR restrictions. Requests to access the datasets should be directed to stijn.vissers@vub.be.

## Ethics statement

The studies involving human participants were reviewed and approved by the Medical Ethics Committee of the Brussels University Hospital and Vrije Universiteit Brussel (B.U.N. 143201939962; 24 March 2019). The patients/participants provided their written informed consent to participate in this study.

## Author contributions

SD, LD, JC, and KC were responsible for the study's conception and design. SV and SD were responsible for data collection and analysis. SV drafted the manuscript. SV, JC, and KC act as guarantors of the work. All authors contributed to the interpretation of the data critically revised the manuscript for important intellectual content and gave final approval for submission.
